# The impact of musculoskeletal pain and strenuous work on self-reported physical work ability: a cohort study of Swedish men and women

**DOI:** 10.1007/s00420-021-01816-6

**Published:** 2021-11-26

**Authors:** Kathryn Badarin, Tomas Hemmingsson, Lena Hillert, Katarina Kjellberg

**Affiliations:** 1 Unit of Occupational Medicine, Institute of Environmental Medicine, Karolinska Institutet, Stockholm, Sweden; 2 Department of Public Health Sciences, Stockholm University, Stockholm, Sweden; 3 Centre for Occupational and Environmental Medicine, Region Stockholm, Stockholm, Sweden

**Keywords:** Ergonomics, Musculoskeletal disorders, Work performance, Job-exposure matrix, Epidemiology

## Abstract

**Objective:**

We investigated the separate and combined effects of musculoskeletal pain (MSP) and strenuous work (heavy physical workload (PWL)/low-decision authority) on poor physical work ability (WA).

**Methods:**

This study uses baseline data from the 2010 Stockholm Public Health Questionnaire (SPHQ) including 9419 workers with good physical WA. Exposure to PWL and decision authority were estimated using sex-specific job-exposure matrices linked to occupations. Exposures (high/low) were combined with the presence of MSP. Follow-up data on physical WA were taken from the 2014 SPHQ and dichotomised (the responses: “moderate”, “rather poor” and “very poor” indicated poor WA). Logistic regression models calculated sex-specific odds ratios adjusting for age, education and health and lifestyle factors. Interaction between MSP and strenuous work was examined using the synergy index (SI). Analyses were conducted using SPSS.27.

**Results:**

MSP, heavy PWL and low-decision authority were separately associated with poor WA. MSP was associated with higher odds of poor WA than strenuous work for women, the opposite for men. Combinations of MSP and strenuous work often resulted in higher risks of poor WA than when adding the effects of the single exposures (e.g., MSP and heavy PWL men: AOR 4.04 95% CI 2.00–8.15, women: AOR: 3.25 95% CI 1.81–5.83). The SI was non-significant for both sexes.

**Conclusion:**

Workers with MSP and strenuous work often had higher risks of poor WA than would be expected from adding the effects of the single exposures. To decrease poor WA in this group, strenuous work should be lowered, and MSP addressed in workplaces.

## Introduction

Musculoskeletal pain (MSP) is widespread among the European workforce and likely to become more prevalent as the number of older workers increases (EU-OSHA 2020). MSP can restrict individuals’ functional capacity and lead to labour market exit (van Rijn et al. 2014). However, many workers with MSP retain good levels of work ability and remain active in the workforce (Pensola et al. 2016). To understand how labour market participation among workers with MSP can be maintained, knowledge of workplace factors associated with work ability for this group is required.

Work ability is a multifaceted concept that encompasses the balance between a workers’ physical and psychological functional capacity and the demands of their job (de Zwart et al. 2002). Poor self-reported work ability has high predictive importance for labour market exit (Alavinia et al. 2009; Lundin et al. 2016). Work ability is usually measured using self-report tools of which the ‘Work Ability Index’ (WAI) is the most common. The WAI is designed to measure different aspects of work ability in relation to work demands, a worker’s health status and mental resources (Ilmarinen 2006).

Multiple individual (e.g., age, obesity, education, leisure-time physical activity and poor musculoskeletal capacity) and work-related factors have been associated with work ability (van Den Berg et al. 2009). Ilmarinen et al. (2005) suggest health and work demands have the largest effect on work ability (Ilmarinen et al. 2005). Specifically, MSP has been associated with poor work ability in several cross-sectional (Bayattork et al. 2019; Miranda et al. 2010; Phongamwong and Deema 2015) and longitudinal (Hallman et al. 2019; Tuomi et al. 1991) studies. High physical workload (PWL) has been associated with poor work ability among workers without (Alavinia et al. 2007, van Den Berg et al. 2009) and with MSP (Oliv et al. 2017; Pensola et al. 2016; Skovlund et al. 2020). Psychosocial work factors, such as job control, have been associated with work ability in some studies (Feldt et al. 2009, van Den Berg et al. 2009) but not others (Gamperiene et al. 2008; Pensola et al. 2016; Vries et al. 2013).

A few studies have explored whether the effect of MSP on work ability may differ between workers with and without strenuous work (Bayattork et al. 2019; Nabe-Nielsen et al. 2014; Neupane et al. 2013). A Danish study found associations between increasing intensity of MSP and poor work ability for workers in sedentary and physically active jobs (Bayattork et al. 2019). Slightly greater risks were found among workers with more physically active jobs. A cross-sectional study of middle-aged employees found separate associations between MSP or physical job demands and reduced work ability, but an interaction between the exposures on work ability was not found (Nabe-Nielsen et al. 2014). Only one longitudinal study exploring the separate and combined effects of MSP and strenuous work on work ability has been found (Neupane et al. 2013). It reported that MSP and awkward postures were separately associated with poor work ability among food industry employees. However, combined exposure to MSP and awkward postures was not associated with higher risks than when adding the effects of the single exposures. In fact, MSP was associated with a higher risk of poor work ability than strenuous work or the combination of MSP and strenuous work.

Some methodological weaknesses in the aforementioned studies should be noted. First, because most of the studies are cross-sectional (Bayattork et al. 2019; Feldt et al. 2009; Gamperiene et al. 2008; Miranda et al. 2010; Nabe-Nielsen et al. 2014; Oliv et al. 2017; Pensola et al. 2016; Phongamwong and Deema 2015), the ability to make causal interpretations about the separate or combined effects of MSP and strenuous work on poor work ability is limited. Second, the studies used self-reported measures to estimate work-related exposures, which could engender a bias due to self-report, particularly among workers with MSP (Gupta et al. 2018). Last, the effects of exposure to workplace factors on musculoskeletal health can manifest differently among men and women (Arbetsmiljöverket 2013, Fillingim 2000). Yet only two aforementioned studies (Oliv et al. 2017; Tuomi et al. 1991) explored sex-specific associations between MSP or strenuous work and work ability.

In this study, we hypothesised that high PWL or low-decision authority aggravates the effect of MSP on the risk of poor work ability. Therefore, we investigated the separate and combined effects of MSP and strenuous working conditions [measured using Job-Exposure Matrices (JEMs)] on poor later self-reported physical WA, separately for men and women.

## Methods

### Participants and study design

**Fig. 1:**
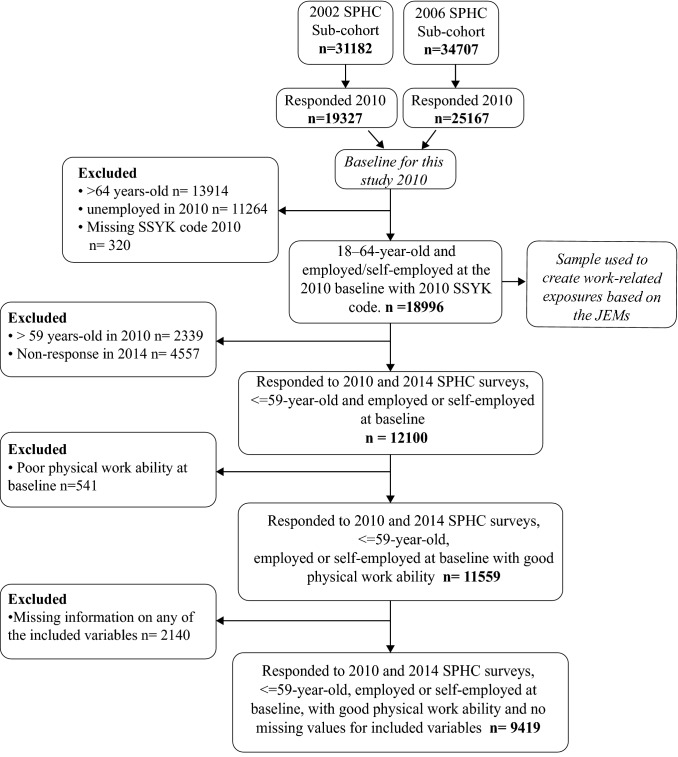
Sample selection. *JEM* job-exposure matrix,* SPHC* Stockholm Public Health Cohort,* SSYK* Swedish Standard Classification of Occupation

This study is based on data from the Stockholm Public Health Cohort (SPHC). The SPHC consists of a random sample of people in Stockholm County that responded to repeated questionnaires. Details of the SPHC are described previously (Svensson et al. 2013). The SPHC has multiple sub-cohorts with different baselines starting at 2002. The present study required data on physical work ability which was only available from follow-up questionnaires in 2010 and 2014. Therefore, for this study, respondents to the 2010 questionnaire from two sub-cohorts (2002 and 2006) were combined to create the baseline sample (Fig. 1). Follow-up data were taken from the 2014 questionnaire. Of the 65,889 respondents to either the 2002 (*n* = 31,182) or 2006 (*n* = 34,707) baseline questionnaires, 44,494 completed the 2010 questionnaire (this study’s baseline) and 32,269 responded to both the 2010 and 2014 questionnaires, an overall response rate of 49%.

Our final sample included employed or self-employed persons likely to not have taken age retirement by 2014 (≤ 59 years old in 2010), with good baseline physical WA, and no missing values for any of the chosen variables (*n* = 9419) (Fig. 1). Good baseline physical work ability was determined by a response of “very good” or “rather good” the question “How do you rate your current work ability in relation to the physical demands of your job?” from the 2010 questionnaire.

### Musculoskeletal pain (exposure)

A dichotomous variable was created to determine the presence of baseline MSP. A case of MSP was determined by a “yes” response to at least one of three questions in the 2010 SPHC questionnaire: “Have you had any pain in the in the past six months in the (i) upper back or neck, (ii) shoulders or arms or iii) lower back?”. Each question had three response categories: “no”; “yes, a few times in the past month or less” or “yes, a few times in the past week or more”.

### Physical workload (exposure)

Baseline exposure to PWL was estimated using a Swedish JEM. The construction of the JEM has been described previously (Badarin et al. 2021). The JEM was developed using responses to eight questions on physical strenuous work from the biennial Swedish Work Environment Surveys (SWES) between 1997 and 2013. Exposure to eight aspects of PWL (heavy lifting (≥ 15 kg), physically strenuous work, fast breathing due to PWL, forward bent position, twisted position, working with hands above shoulder level, repetitive work and frequent bending and twisting) were calculated using a 1-to-5/6-point rating scale (1 = lowest and 5/6 = highest exposure). An index score (overall PWL) was created by summing the scores for each of the eight PWL exposures and calculating a mean value. The JEM provides gender-specific arithmetic mean values for 355 different occupations, coded with the Swedish Standard Classification of Occupation (SSYK) 96 coding system. The SSYK 96 codes (occupational titles) for the 2010 baseline participants were obtained from the Longitudinal Integration Database for Health Insurance and Labor Market Studies (LISA) linked to SPHC.

In this study, the mean JEM values for four PWL exposures; overall PWL, heavy lifting (≥ 15 kg), working in a forward bent position and fast breathing due to PWL, were assigned to the SSYK codes of all SPHC participants with an SSYK code, 18–64 years old and employed/self-employed at the 2010 baseline (*n* = 18,996), before the exclusion of those older than 59 years (Fig. 1). Gender-specific dichotomous variables were created using a median cut-off (< median = low PWL and ≥ median = high PWL).

### Decision authority (exposure)

Baseline exposure to decision authority at work was estimated using a Swedish JEM for psychosocial workload. The JEM was developed on the same material and with the same procedure as the physical JEM and has been previously described (Almroth et al. 2021). The JEM provides a gender-specific mean index score for decision authority based on responses to four questions on perceived control over when tasks are conducted, work pace, work breaks and work structure. The JEM scores are linked to occupations using the SSYK 96 coding system. The index scores for decision authority were fixed to 2010 baseline SSYK codes (from LISA) for each participant in this study, based on the same sample of 18,996 workers used for the PWL exposures. Sex-specific binary variables were created using the median as a cut-off (> median = high decision authority and ≤ median = low-decision authority).

### Poor self-reported physical work ability (outcome)

Physical work ability was defined by a single item from the WAI included in the 2014 SPHC questionnaire: “How do you rate your current work ability in relation to the physical demands of your job?” with five responses options: “very good”, “rather good”, “moderate”, “rather poor”, and “very poor”. Less than “rather good” indicated poor physical work ability. The physical work ability item has shown a strong correlation with the full WAI (Ebener and Hasselhorn 2019), performed well at predicting sick leave (Vingard et al. 2005), and used to explore associations between MSP or strenuous work and work ability in previous studies (Skovlund et al. 2020; Bayattork et al. 2019).

### Covariates

Potential confounders were identified from the literature.

### Completed level of education

Data on completed level of education were taken from LISA. A categorical variable indicated three groups primary (≤ 9 years), secondary (10–12 years) and tertiary (> 12 years) education.

The following covariates were from the 2010 SPHC questionnaire.

### Psychological distress

The 12-item General Health Questionnaire (GHQ12) was used to estimate psychological distress. The scores for the GHQ12 range from 0 to 12. A binary variable was created using ≥ 3 to signify psychological distress (Banks 1980). The GHQ12 has been shown to be a reliable and valid scoring measure to predict common mental illnesses (Petkovska et al. 2015).

### Long term health conditions

A “yes” response to the question: “Do you suffer from a long-term illness, health problems following an accident, disability or other persistent health problems?” indicated the presence of a long-term health condition.

### Body mass index (BMI)

BMI [weight(kg)/(height × height)(m^2^)] was calculated using data from the SPHC. A categorical variable was created with three groups: underweight and normal weight (BMI < 25), overweight (BMI ≥ 25 and BMI < 30), and obese (BMI ≥ 30).

### Smoking

Participants were asked “Do you currently smoke daily?” A binary variable yes/no indicated being a current smoker.

### Leisure-time physical activity

Two questions on leisure-time physical activity were posed. The first question “Average daily amount of walking/cycling over the past 12 months”, had the response options: “almost never”; “less than 20 min a day”; “20–40 min a day”; “40–60 min per day”; “1–1.5 h per day” or “more than 2 h a day. The second question “Average weekly amount of physical activity other than walking/cycling” had the response options: “almost never”; “less than 1 h per week”; “1–2 h per week”; “2–3 h per week”; “3–4 h per week”; “4–5 h per week”; “more than 5 h per week”. Responses to both questions were coded as minutes and combined to create an overall estimate of weekly leisure-time physical activity. A final binary variable was created based on the World Health Organization’s recommendation of 150 min of moderate-intensity aerobic physical activity a week for adults between ages 18–64 (World Health Organization 2004) (≥ 150 min and < 150 min weekly leisure-time physical activity).

### Statistical analysis

All statistical analyses were conducted using SPSS version 25.0. First, univariate associations between all covariates and poor work ability were assessed separately for men and women using logistic regression analysis. Second, logistic regression models were used to estimate the associations between combinations of MSP and strenuous work for the risk of poor physical WA, with those jointly unexposed as the reference category: (i) no MSP and non-strenuous work (reference category) (ii) no MSP and strenuous work (iii) MSP and non-strenuous work and (iv) MSP and strenuous work. Crude (OR) and adjusted odds ratios (AOR) were computed for men and women, with 95% confidence intervals (95% CI). Confounding effects of the covariates on the main exposures were explored by grouping covariates according to health and lifestyle factors (model 1), work factors (model 2) and education (model 3). All analyses were adjusted for age. Because educational attainment evidently affects the selection into occupations its inclusion as a confounder could cause over adjustment (34). Therefore, a fully adjusted model excluding education (model 4) and a fully adjusted model (model 5) were added. Confounders were chosen based on their empirical and theoretical significance with the exposure and outcome.

Interaction effects between MSP and strenuous work were explored using the synergy index (SI) first presented by Rothman (1986). In this study, the SI measures the extent to which the effect of combined exposure to MSP and heavy PWL or low-decision authority on poor work ability exceeds the sum of the effects of each exposure separately when those unexposed to both exposures are used as reference category (VanderWeele and Knol 2014; Andersson et al. 2005). The SI is defined as:

$${\text{SI}} = \frac{{RR_{11} - 1}}{{\left( {RR_{10} - 1} \right) + \left( {RR_{01} - 1} \right)}}.$$

If the SI > 1 implies there is a synergistic interaction. The 95% CI for the SI were calculated according to (Andersson et al. (2005).

## Results

Of the 3911 male and 5508 female employees with good work ability in 2010, 161 cases of poor work ability were observed among men and 322 among women after the 4 year follow-up. MSP and more severe levels of MSP were more prevalent among women than men (Appendix 1).

### Distribution of covariates in categories of PWL and decision authority

The proportions of workers above 50 years old, with low education, who smoked daily, were overweight/obese or with less than 150 min of leisure-time physical activity per week was higher among those with high compared to low PWL, for both sexes (Table 1). The opposite was observed for psychological distress.

**Table 1:** Prevalence of covariates in different PWL and decision authority categories among men and women

	Baseline characteristics		Physical workload				Decision Authority					
			Low		High		High		Low		Total	
			*n*	%	*n*	%	*n*	%	*n*	%	*n*	%
Men *n* = 3911	Age	18–29	67	3,1	105	6,1	73	3,3	99	5,9	172	4,4
		30–39	601	27,6	356	20,5	572	25,6	385	23,0	957	24,5
		40–49	817	37,5	633	36,5	844	37,7	606	36,2	1450	37,1
		50–59	692	31,8	640	36,9	748	33,4	584	34,9	1332	34,1
	Completed Education^a^	> 12	1628	74,8	680	39,2	1561	69,8	747	44,6	2308	59,0
		10–12	507	23,3	854	49,3	592	26,5	769	45,9	1361	34,8
		< *9*	42	1,9	200	11,5	84	3,8	158	9,4	242	6,2
	Health condition	No	1732	79,6	1342	77,4	1768	79,0	1306	78,0	3074	78,6
		Yes	445	20,4	392	22,6	469	21,0	368	22,0	837	21,4
	Psychological distress^b^	No	1821	83,6	1514	87,3	1882	84,1	1453	86,8	3335	85,3
		Yes	356	16,4	220	12,7	355	15,9	221	13,2	576	14,7
	Smoking	No	2086	95,8	1571	90,6	2131	95,3	1526	91,2	3657	93,5
		Yes	91	4,2	163	9,4	106	4,7	148	8,8	254	6,5
	BMI	Underweight/normal	754	34,6	477	27,5	746	33,3	485	29,0	1231	31,5
		Overweight	1239	56,9	1050	60,6	1303	58,2	986	58,9	2289	58,5
		Obese	184	8,5	207	11,9	188	8,4	203	12,1	391	10,0
	Leisure physical activity	≥ 150 min	1871	85,9	1380	79,6	1904	85,1	1347	80,5	3251	83,1
		< 150 min	306	14,1	354	20,4	333	14,9	327	19,5	660	16,9
Women *n* = 5508	Age	18–29	140	4,6	173	7,0	151	4,9	162	6,7	313	5,7
		30–39	824	27,1	544	22,1	822	26,5	546	22,7	1368	24,8
		40–49	1154	37,9	898	36,5	1233	39,7	819	34,1	2052	37,3
		50–59	928	30,5	847	34,4	900	29,0	875	36,4	1775	32,2
	Completed Education^a^	> 12	2275	74,7	1190	48,3	2103	67,7	1362	56,7	3465	62,9
		10–12	708	23,2	1144	46,5	906	29,2	946	39,4	1852	33,6
		< *9*	63	2,1	128	5,2	97	3,1	94	3,9	191	3,5
	Health condition	No	2393	78,6	1941	78,8	2444	78,7	1890	78,7	4334	78,7
		Yes	653	21,4	521	21,2	662	21,3	512	21,3	1174	21,3
	Psychological distress^b^	No	2445	80,3	2023	82,2	2500	80,5	1968	81,9	4468	81,1
		Yes	601	19,7	439	17,8	606	19,5	434	18,1	1040	18,9
	Smoking	No	2867	94,1	2192	89,0	2898	93,3	2161	90,0	5059	91,8
		Yes	179	5,9	270	11,0	208	6,7	241	10,0	449	8,2
	BMI	Underweight/normal	1756	57,6	1273	51,7	1776	57,2	1253	52,2	3029	55,0
		Overweight	1059	34,8	924	37,5	1084	34,9	899	37,4	1983	36,0
		Obese	231	7,6	265	10,8	246	7,9	250	10,4	496	9,0
	Leisure physical activity	≥ 150 min	2752	90,3	2161	87,8	2798	90,1	2115	88,1	4913	89,2
		< 150 min	294	9,7	301	12,2	308	9,9	287	11,9	595	10,8

*PWL* physical workload

^a^ > 12 = tertiary; 10–12 = secondary; ≤ 9 = primary

^b^Yes = GHQ12 > 3

Among both sexes with low-decision authority, the proportions of workers with low education, who smoked daily, were obese or with < 150 min of leisure-time physical activity per week was larger than among those with high decision authority. The opposite was observed for psychological distress.

### Association between covariates and poor physical work ability

The univariate analyses showed statistically significant associations between completed education (primary or secondary), long-term health conditions, psychological distress, smoking, being obese and < 150 min leisure-time physical activity per week and poor work ability for both sexes (Table 2).

**Table 2:** Bivariate associations between covariates and poor physical work ability

Baseline characteristics		Men *n* = 3911		Women *n* = 5508
		OR poor work ability at follow-up		OR of poor work ability at follow-up
		OR (95% CI)		OR (95% CI)
Completed Education^a^	> 12	1		1
	10–12	2,28 (1,62–3,21)		1,87 (1,48–2,36)
	< 9	3,91 (2,39–6,49)		2,82 (1,76–4,52)
Health condition	No	1		1
	Yes	2,09 (1,50–2,91)		2,33 (1,84–2,95)
Psychological distress^b^	No	1		1
	Yes	1,91 (1,32–2,77)		1,62 (1,25–2,09)
Smoking	No	1		1
	Yes	2,01 (1,22–3,30)		1,55 (1,09–2,21)
BMI	Underweight/ normal	1		1
	Overweight	1,34 (0,92–1,95)		1,48 (1,16–1,88)
	Obese	2,18 (1,31–3,63)		1,85 (1,30–2,65)
Leisure physical activity	≥ 150 min	1		1
	< 150 min	1,85 (1,30–2,65)		1,86 (1,38–2,51)

*OR* odds ratios

95% CI 95% confidence intervals

^a^ > 12 = tertiary, 10–12 = secondary, ≤ 9 = primary

^b^Yes = GHQ12 > 3

### Separate and combined effects of MSP and strenuous work on poor physical WA

Compared to workers without MSP and with non-strenuous work, separate exposure to MSP (e.g., men: OR 2.14 95% CI: 1.05–4.34, women: OR 3.01 95% CI 1.71–5.28), high PWL (e.g., overall heavy PWL men: OR 3.94, 95% CI 1.90–8.18, women: OR 2.39 95% CI 1.22–4.65) or low-decision authority (men: OR 1.98 95% CI 1.04–3.78, women: OR 2.47 95% CI 1.26–4.81) were associated with a statistically significant increased relative risk of poor work ability (Tables 3 and 4).

**Table 3:** Crude and adjusted odds ratios (OR with 95 confidence intervals (CI95)) of poor physical work ability at follow-up according to baseline MSP, physical workload/decision authority and their combinations among male workers with good baseline work ability (*n* = 3911)

		Cases/*n*	Crude	Model 1	Model 2	Model 3	Model 4	Model 5
MSP/ heavy physical work	No/no	10/823	1	1	1	1	1	1
	No/yes	29/595	3,94(1,90–8,18)	3,79(1,82–7,89)	3,37(1,59–7,13)	3,38(1,61–7,09)	3,26(1,54–6,93)	2,95(1,38–6,31)
	Yes/no	36/1354	2,14(1,05–4,34)	1,86(0,91–3,79)	2,14(1,05–4,33)	2,09(1,03–4,25)	1,87(0,92–3,82)	1,85(0,91–3,77)
	Yes/yes	86/1139	6,13(3,16–11,89)	5,36(2,75–10,47)	5,14(2,58–10,24)	5,11(2,59–10,08)	4,56(2,28–9,11)	4,04(2,00–8,15)
SI (95%CI)			1,35 (0,79–2,28)	1,30 (0,73–2,30)	1,26 (0,73–2,18)	1,27 (0,74–2,19)	1,22 (0,67–2,21)	1,16 (0,62–2,18)
MSP/ heavy lifting	No/no	11/846	1	1	1	1	1	1
	No/yes	28/572	3,75 (1,85–7,62)	3,57(1,75–7,29)	3,17(1,54–6,56)	3,18(1,55–6,55)	3,04(1,46–6,32)	2,73 (1,30–5,72)
	Yes/no	39/1380	2,14 (1,09–4,20)	1,85(0,94–3,66)	2,14(1,09–4,22)	2,09 (1,06–4,12)	1,87(0,95–3,70)	1,85(0,94–3,66)
	Yes/yes	83/1113	5,69 (3,01–10,76)	4,97(2,61–9,47)	4,70(2,43–9,10)	4,71(2,44–9,06)	4,16(2,14–8,09)	3,66(1,86–7,21)
SI (95%CI)			1,29 (0,76–2,20)	1,26 (0,71–2,26)	1,19 (0,68–2,08)	1,21 (0,69–2,12)	1,16 (0,60–2,13)	1,10 (0,58–2,09)
MSP/ forward bent position	No/no	11/843	1	1	1	1	1	1
	No/yes	28/575	3,69(1,82–7,49)	3,44 (1,68–7,02)	3,16(1,51–6,62)	3,16 (1,54–6,46)	2,95 (1,41–6,19)	2,69(1,28–5,66)
	Yes/no	38/1384	2,06(1,04–4,05)	1,79(0,90–3,54)	2,05(1,04–4,05)	1,99 (1,01–3,92)	1,80 (0,91–3,56)	1,76(0,89–3,48)
	Yes/yes	84/1109	5,76 (3,04–10,88)	4,94(2,60–9,39)	4,87(2,48–9,57)	4,81(2,52–9,21)	4,19 (2,13–8,24)	3,74(1,89–7,40)
SI (95%CI)			1,33 (0,78–2,78)	1,28 (0,72–2,29)	1,25 (0,71–2,21)	1,27 (0,73–2,23)	1,16 (0,61–2,22)	1,16 (0,61–2,23)
MSP/ Fast breathing	No/no	8/868	1	1	1	1	1	1
	No/yes	31/550	5,99 (2,73–13,17)	5,51 (2,50–12,17)	5,27 (2,33–11,96)	5,12 (2,31–11,39)	4,84 (2,12–11,04)	4,32 (1,88–9,92)
	Yes/no	40/1395	3,06 (1,42–6,57)	2,63 (1,22–5,67)	3,05 (1,42–6,56)	2,98 (1,39–6,41)	2,63 (1,22–5,67)	2,58 (1,20–5,57)
	Yes/yes	82/1098	7,92 (3,81–16,50)	6,84 (3,27–14,34)	6,93 (3,21–14,97)	6,65 (3,15–14,05)	5,98 (2,75–12,99)	5,25 (2,39–11,52)
SI (95%CI)			1,03 (0,66–1,60)	1,00 (0,63–1,59)	0,97 (0,33–2,91)	0,97 (0,33–2,88)	0,94 (0,58–1,53)	0,90 (0,54–1,48)
MSP/low decision authority	No/no	17/838	1	1	1	1	1	1
	No/yes	22/580	1,98 (1,04–3,78)	1,80(0,94–3,46)	1,29(0,66–2,51)	1,70(0,89–3,26)	1,20 (0,62–2,35)	1,16 (0,59–2,28)
	Yes/no	44/1399	1,56 (0,88–2,75)	1,32(0,75–2,35)	1,57(0,89–2,78)	1,51(0,85–2,66)	1,35(0,76–2,40)	1,33(0,75–2,36)
	Yes/yes	78/1094	3,59 (2,10–6,13)	3,08(1,79–5,29)	2,28(1,30–4,00)	2,97(1,72–5,13)	1,99(1,13–3,51)	1,89(1,07–3,34)
SI (95%CI)			1,84 (0,78–4,34)	1,96 (0,68–5,63)	1,59 (0,61–4,14)	1,27 (0,74–2,19)	1,81 (0,36–9,19)	1,84 (0,30–11,31)

*SI* synergy index; *MSP* musculoskeletal pain; all analyses adjusted for age

Model 1: smoking, long-term health condition, BMI, psychological distress and leisure-time physical activity

Model 2: decision authority/ PWL index

Model 3: education

Model 4: model 1 + model 2

Model 5: full model

**Table 4:** Crude and adjusted odds ratios (OR with 95 confidence intervals (CI95)) of poor physical work ability at follow-up according to baseline MSP, physical workload/decision authority and their combinations among female workers with good baseline work ability (*n* = 5508)

		Cases/*n*	Crude	Model 1	Model 2	Model 3	Model 4	Model 5
MSP/ heavy physical work	No/no	14/830	1	1	1	1	1	1
	No/yes	24/605	2,39 (1,22–4,65)	2,39 (1,22–4,68)	1,75 (0,88–3,46)	2,10 (1,07–4,11)	1,77 (0,89–3,51)	1,51 (0,76–3,02)
	Yes/no	107/2216	3,01 (1,71–5,28)	2,58 (1,47–4,55)	2,99 (1,70–5,25)	2,94 (1,68–5,17)	2,56 (1,45–4,51)	2,48 (1,41–4,38)
	Yes/yes	177/1857	6,20 (3,57–10,75)	5,25 (3,01–9,13)	4,50 (2,55–7,98)	5,40 (3,10–9,42)	3,83 (2,15–6,80)	3,25 (1,81–5,83)
SI (95% CI)			1,57 (1,02–2,43)	1,48 (1,92–2,38)	1,33 (0,82–2,14)	1,49 (0,94–2,37)	1,26 (0,78–2,03)	1,18(0,68–2,07)
MSP/ heavy lifting	No/no	11/816	1	1	1	1	1	1
	No/yes	27/619	3,37 (1,66–6,85)	3,42 (1,68–6,97)	2,63 (1,28–5,41)	3,12 (1,53–6,35)	2,69 (1,31–5,56)	2,49 (1,21–5,16)
	Yes/no	97/2187	3,48 (1,86–6,53)	2,99 (1,59–5,62)	3,44 (1,83–6,46)	3,42 (1,82–6,42)	2,95 (1,57–5,56)	2,87 (1,53–5,41)
	Yes/yes	187/1886	8,20 (4,44–15,15)	7,00 (3,78–12,97)	6,38 (3,41–11,97)	7,44 (4,02–13,77)	5,48 (2,91–10,31)	5,02 (2,66–9,47)
SI (95% CI)			1,51 (1,02–2,23)	1,40 (0,92–2,13)	1,35 (0,90,2,03)	1,46 (0,98–2,16)	1,26 (0,82–1,95)	1,24 (0,79–1,94)
MSP/ forward bent position	No/no	13/807	1	1	1	1	1	1
	No/yes	25/628	2,52 (1,28–4,97)	2,54 (1,29–5,02)	1,73 (0,85–3,54)	2,39 (1,21–4,71)	1,80 (0,88–3,69)	1,74 (0,85–3,57)
	Yes/no	103/2176	3,09 (1,72–5,53)	2,60 (1,45–4,68)	3,04 (1,70–5,46)	3,00 (1,68–5,38)	2,56 (1,43–4,61)	2,47 (1,37–4,45)
	Yes/yes	181/1897	6,53 (3,70–11,54)	5,60 (3,16–9,93)	4,49 (2,44–8,27)	6,07 (3,43–10,74)	3,96 (2,14–7,32)	3,79 (2,05–7,00)
SI (95% CI)			1,57 (1,03–2,39)	1,52 (0,94–2,45)	1,30 (0,81–2,09)	1,55 (1,00–2,41)	1,33 (0,76–2,32)	1,26 (0,82–1,95)
MSP/ Fast breathing	No/no	11/784	1	1	1	1	1	1
	No/yes	27/651	3,04 (1,50–6,18)	3,08 (1,51–6,28)	2,31 (1,12–4,79)	2,89 (1,42–5,88)	2,37 (1,14–4,92)	2,28 (1,10–4,74)
	Yes/no	99/2185	3,41 (1,82–6,39)	2,89 (1,54–5,44)	3,37 (1,80–6,32)	3,33 (1,78–6,25)	2,85 (1,51–5,36)	2,77 (1,47–5,21)
	Yes/yes	185/1888	7,73 (4,18–14,29)	6,63 (3,57–12,28)	5,80 (3,07–10,97)	7,17 (3,87–13,27)	5,03 (2,65–9,55)	4,77 (2,51–9,06)
SI (95% CI)			1,55 (1,05–2,30)	1,47 (0,95–2,28)	1,35 (0,88–2,07)	1,52 (1,01–2,27)	1,31 (0,83–2,08)	1,31 (0,81–2,12)
MSP/low decision authority	No/no	14/843	1	1	1	1	1	1
	No/yes	24/592	2,47 (1,26–4,81)	2,52 (1,29–4,92)	1,96 (0,99–3,88)	2,35 (1,21–4,59)	2,02 (1,02–4,01)	2,07 (1,04–4,12)
	Yes/no	105/2263	2,93 (1,67–5,15)	2,54 (1,44–4,48)	2,91 (1,66–5,12)	2,87 (1,63–5,04)	2,53 (1,44–4,46)	2,47 (1,40–4,36)
	Yes/yes	179/1810	6,51 (3,76–11,29)	5,54 (3,18–9,63)	5,14 (2,90–9,09)	6,07 (3,50–10,54)	4,40 (2,48–7,81)	4,48 (2,52–7,95)
SI (95% CI)			1,62 (1,04–2,51)	1,50 (0,94–2,42)	1,43 (0,86–2,37)	1,49 (0,94–2,37)	1,35 (0,82–2,23)	1,40 (0,84–2-33)

*SI* synergy index; *MSP* musculoskeletal pain; all analyses adjusted for age

Model 1: smoking, long-term health condition, BMI, psychological distress and leisure-time physical activity

Model 2: decision authority/PWL index

Model 3: education

Model 4: model 1 + model 2

Model 5: full model

Separate exposure to high PWL (overall PWL, heavy lifting (≥ 15 kg), forward bent position or fast breathing) was associated with a larger relative risk of poor work ability than separate exposure to MSP or low-decision authority for men. Among women, MSP was associated with a larger relative risk of poor work ability than heavy PWL or low-decision authority.

Workers with combined exposed to MSP and strenuous work (e.g., overall heavy PWL and MSP, men: OR 6.13 95% CI, 3.16–11.89, women: OR 6.20 95% CI, 3.57–10.75) had higher risks of poor work ability than when adding the effects of the single exposures (Tables 3 and 4). The *SI* was non-statistically significant for men (Table 4) but statistically significant for women (e.g., MSP and overall PWL SI = 1.57 95% CI 1.02–2.43) (Table 3).

After adjusting for age, education, smoking, long-term health condition, BMI, psychological distress, leisure-time physical activity and decision authority/heavy PWL, most associations between MSP and poor work ability became non-statistically significant among men [apart from when compared to workers without MSP and not exposed to with physical work causing fast breathing, AOR 2.58 95% CI 1.20–5.57)]. For women, all associations between MSP and poor work ability remained statistically significant (e.g., AOR 2.48 95% CI 1.41–4.38).

Separate exposure to all investigated aspects of heavy PWL remained statistically significantly associated with poor work ability for men (e.g., overall heavy PWL men: AOR 2.95 95% CI 1.38–6.31). For women, statistically significant relative risk of poor work ability remained for heavy lifting (AOR 2.49 95% CI 1.21–5.16) and fast breathing (AOR 2.28 95% CI 1.10–4.74). Low decision authority was statistically significantly associated with poor work ability for women (AOR 2.07 95% CI 1.04–4.12), but not men.

All adjusted relative risks for combined exposure to MSP and strenuous work remained statistically significant for both sexes (e.g., MSP and overall heavy PWL men: AOR 4.04 95% CI 2.00–8.15 women: 3.25 95% CI 1.81–5.83). However, the adjusted estimates for the *SI* were non-statistically significant.

## Discussion

### Summary of the findings

To our knowledge, this is the first prospective study to explore the separate and combined effects of MSP and strenuous work (high PWL or low-decision authority) on poor self-reported physical work ability among men and women in the general working population.

MSP, heavy PWL and low-decision authority were separately associated with poor self-reported physical work ability among both sexes. Workers with combined exposure to MSP and heavy PWL or low-decision authority often had higher risks of poor WA than when adding the effects of the single exposures e.g., the SI were often higher than 1. This finding indicates that the relationship between PWL and MSP on poor work ability was more than additive and shows support for the hypothesis that strenuous work aggravates the effect of MSP on poor work ability. However, the estimates for SI were only statistically significant for women. After adjustments, all associations between MSP and poor work ability remained for women, but only one remained for men. Exposure to heavy PWL remained associated with poor work ability among both sexes, but more consistent associations were found among men. Separate exposure to low-decision authority was only associated with poor work ability among women. The relative risks for the combined effects of MSP and strenuous work on poor work ability remained greater than the sum of the individual effects, but the estimates for the SI were not statistically significant.

### Comparison with previous studies

Our finding that separate exposure to MSP was associated with an increased risk of poor self-reported work ability is in accordance with the results of several existing studies (Bayattork et al. 2019; Miranda et al. 2010; Phongamwong and Deema 2015; Hallman et al. 2019; Tuomi et al. 1991), most of them cross-sectional (Bayattork et al. 2019; Miranda et al. 2010; Phongamwong and Deema 2015). Our findings strengthen the current evidence by showing a prospective association between MSP and poor physical work ability.

Our findings also support the results from previous studies showing associations between heavy PWL and work ability for workers without (Alavinia et al. 2007, van Den Berg et al. 2009) and with MSP (Oliv et al. 2017; Pensola et al. 2016; Skovlund et al. 2020). We found stronger associations between heavy PWL and poor work ability among men than women. Only a few existing studies have explored sex-specific associations between PWL and work ability. A Swedish study on workers with neck pain observed stronger associations between low exposure to physical work demands (lifting, twisted work posture, working with hands in shoulder level or higher, and forward bending) and excellent work ability for men than women (Oliv et al. 2017). A Dutch study on male construction workers found associations between awkward postures or manual handling tasks and poor work ability (Alavinia et al. 2007), whereas a Norwegian study on female workers did not observe an association between self-reported level of overall strenuous work and poor work ability (Gamperiene et al. 2008). In fact, like our results, the Norwegian study showed stronger associations between poor self-reported physical health and poor work ability than strenuous work conditions. Overall, our findings and the results of the aforementioned studies allude to a potential difference in the relationship between PWL and poor work ability for men and women.

In this study, low-decision authority was associated with poor physical work ability among women, but not among men. Earlier studies exploring job control and work ability have shown discordant results, some finding an association (Feldt et al. 2009, van Den Berg et al. 2009) and some not (Gamperiene et al. 2008; Pensola et al. 2016). Interestingly, in contrast to our results, the aforementioned study on Norwegian female workers did not find associations between job control and poor work ability (Gamperiene et al. 2008). The differing findings may be a result of methodological differences, such as exploration of different samples and varied measures of control.

One earlier prospective study exploring the separate and combined effects of MSP and strenuous work on poor work ability has been found (Neupane et al. 2013). The study of food industry employees showed separate associations between the presence of multisite MSP and exposure to strenuous work (awkward postures) and poor work ability. MSP was associated with a higher risk of poor work ability than strenuous work, which is consistent with our results for women. The study did not conduct sex-stratified analysis, however, 65% of their sample were women. In contrast to our findings, the study (Neupane et al. 2013) did not find more than additive effects. In fact, single exposure to MSP was associated with a higher relative risk of poor work ability than combined exposure to MSP and strenuous work. A cross-sectional study exploring associations between increasing intensity of MSP and poor work ability found greater risks of poor work ability among workers with physically active jobs compared to sedentary jobs (Bayattork et al. 2019), but the difference between the OR in the two groups was not statistically significant. A cross-sectional study on middle-aged employees did not find an interaction between MSP and strenuous work on poor work ability (Nabe-Nielsen et al. 2014).

### Strengths and limitations

A strength of this study is its longitudinal design. However, response to population-based surveys is often higher among people with advantaged social positions and those with better health (Martikainen et al. 2016). Therefore, nonresponse and attrition bias may have reduced the strength of our estimates and limit our findings generalisability. That said, an exploration of a sample that included non-responders to the 2014 SPHC questionnaire showed few characteristic differences compared to our final sample. The relatively large sample allowed us to conduct sex-stratified analysis and adjust for range of confounding factors. Nevertheless, residual confounding should be considered.

Another strength is the application of the JEMs. The Swedish JEMs have shown good external validity when predicting worsening MSP (Badarin et al. 2021), disability pension (Falkstedt et al. 2021) or diagnosed depression (Almroth et al. 2021). Studies that examine the use of existing JEMs, constructed using similar methods, suggest that the exposure estimates provided by the JEMs can be used to identify occupations with higher risks of MSP and other health outcomes (Rijs et al. 2014, Hanvold et al. 2018; Solovieva et al. 2012). The JEMs allowed us to explore multiple workplace exposures, though, it is hard to disentangle their independent effects as many workplace risk factors are interrelated. It is important to also note some limitations of the JEMS. First, they were created using self-reported data, which is generally perceived as a less accurate measure of PWL than technical measurements e.g., accelerometery (Wells et al. 1997). Nevertheless, self-reported exposure measures are frequently used in large epidemiological studies due to low costs and practicalities. Additionally, because the JEMs were constructed using self-reported data collected from a different sample than the one investigated in this study, the JEMs provide a more independent measure of workplace exposures compared to previous studies that have relied on self-reported data obtained from the same persons. Second, the JEMs provide aggregated estimates of workplace exposures, therefore, the heterogeneity of exposures within occupations is lost. This could lead to non-differential misclassification and an underestimation of the true estimates (Hanvold et al. 2018).

A further limitation is the single baseline exposure measurement, which does not account for exposure changes over time. Additionally, to create enough statistical power to explore combined exposures and produce sex-specific results our definition of MSP was broad. The strength of the effect of MSP on work ability may differ depending on the severity of MSP explored. It should also be noted that the 2010 SPHC did not include questions on MSP in the lower limbs therefore lower limb MSP was not explored in this study.

Work ability is a complex concept to measure, and its operationalisation varies. It is often infeasible to include the full WAI in surveys; therefore, single items (such as the physical work ability applied in this study) that have shown high correlations with the full WAI are used (Ebener and Hasselhorn 2019).

### Interpretation of the results

Our finding that MSP is an important factor contributing to reduced physical WA, either separately or in combination with strenuous work, underscores the need to combat the prevalence of MSP among workers. MSP was associated with a higher risk of reporting poor work ability among women than men. It should be noted that in our sample, MSP was more prevalent among women than men and more women reported severe MSP than men. Biological (e.g., hormonal) and psychosocial differences (e.g., coping strategies and self-efficacy) between men and women have been identified as explanations for sex/gender-based differences in reports of MSP (Sorge and Totsch 2017; Fillingim 2000). Differences in motor control strategies when performing physical work tasks may also cause women to experience more MSP than men (Cid et al. 2019). Overall, these differences suggest that women have a higher vulnerability to MSP than men and could partly explain why we found that MSP had a greater effect on poor work ability among women than men.

Heavy PWL was associated with a larger risk of poor work ability than MSP among men. A Dutch study also found that high physical demands were more strongly associated with work ability than physical health (respiratory/cardiovascular health) among male construction workers (Alavinia et al. 2009). Typically, men are overrepresented in jobs with high exposure to heavy PWL (Arbetsmiljöverket 2013). This was reflected in our sample as men in the high PWL category reported higher levels of PWL than women in the high PWL category. When exposure to heavy PWL is very high it could have a greater impact on people’s self-report of poor work ability than health conditions (e.g., MSP).

In this study, male and female workers with combined exposure to MSP and strenuous work often had higher risks of poor work ability than would be expected from adding each exposure (the SI were often higher than 1). That said, the estimates for the synergy index were only statistically significant for the crude results among women. The additive, and signs of more than additive, effects among the combined exposure group concur with the hypothesis that strenuous work aggravates the effect of MSP on the risk of poor work ability. Reducing exposure to strenuous work appears to be a reasonable approach to lower the risk of poor work ability among workers with combined exposure to MSP and strenuous work. Workers with MSP should have the opportunity to receive support to adjust their work (e.g., environmental adaptations, provision of ergonomic equipment or increased autonomy over the organisation of work tasks) to match a reduction in physical capacity.

### Conclusion

Workers with combined exposure to MSP and strenuous work often had higher risks of poor work ability than would be expected from adding the effects of the single exposures. To decrease the level of poor work ability in this group, exposure to strenuous work should be lowered and MSP should be addressed in workplaces.

## Data Availability

Data may be obtained from a third party and are not publicly available. The data used for this study were obtained from Statistics Sweden (SCB).

## Appendix 1

Distribution of MSP and severity of MSP among level of physical workload or decision authority for men and women.

**Table:**

			Physical workload				Decision Authority				Total	
			Low		High		High		Low			
			*n*	%	*n*	%	*n*	%	*n*	%	*n*	%
Men *n* = 3911	MSP	No pain	823	37,8	595	34,3	838	37,5	580	34,6	1418	36,3
		pain	1354	62,2	1139	65,7	1399	62,5	1094	65,4	2493	63,7
	MSP location	No pain	823	37,8	595	34,3	838	37,5	580	34,6	1418	36,3
		Low back	397	18,2	276	15,9	400	17,9	273	16,3	673	17,2
		Arms and/or Shoulder	135	6,2	112	6,5	141	6,3	106	6,3	247	6,3
		Upper back	170	7,8	121	7,0	165	7,4	126	7,5	291	7,4
		Multisite pain	652	29,9	630	36,3	693	31,0	589	35,2	1282	32,8
	MSP frequency	No pain	823	37,8	595	34,3	838	37,5	580	34,6	1418	36,3
		Monthly	1001	46,0	812	46,8	1026	45,9	787	47,0	1813	46,4
		Weekly	353	16,2	327	18,9	373	16,7	307	18,3	680	17,4
Women *n* = 5508	MSP	No pain	830	27,2	605	24,6	843	27,1	592	24,6	1435	26,1
		pain	2216	72,8	1857	75,4	2263	72,9	1810	75,4	4073	73,9
	MSP location	No pain	830	27,2	605	24,6	843	27,1	592	24,6	1435	26,1
		Low back	315	10,3	258	10,5	303	9,8	270	11,2	573	10,4
		Arms and/or Shoulder	183	6,0	138	5,6	189	6,1	132	5,5	321	5,8
		Upper back	309	10,1	183	7,4	315	10,1	177	7,4	492	8,9
		Multisite pain	1409	46,3	1278	51,9	1456	46,9	1231	51,2	2687	48,8
	MSP frequency	No pain	830	27,2	605	24,6	843	27,1	592	24,6	1435	26,1
		Monthly	1478	48,5	1196	48,6	1511	48,6	1163	48,4	2674	48,5
		Weekly	738	24,2	661	26,8	752	24,2	647	26,9	1399	25,4

*MSP* musculoskeletal pain
